# Trisomy 21 and Assisted Reproductive Technologies: A review

**DOI:** 10.5935/1518-0557.20210047

**Published:** 2022

**Authors:** Edgard Sánchez-Pavón, Hector Mendoza, Javier García-Ferreyra

**Affiliations:** 1 EmbryoFertility Reproductive Biomedicine, Lima, Peru; 2 University of Louisville, USA; 3 Laboratory of Assisted Reproduction. Alcivar Hospital, Guayaquil, Ecuador

**Keywords:** Trisomy 21, down syndrome, aneuploidy, ART

## Abstract

Trisomy 21 is the most common genetic disorder seen among infants, and it causes spontaneous abortions, abnormal neural development and other pathologies associated with newborn development. In newborns with this trisomy, 90-95% have full trisomy, 1.4-1.9% have mosaicism, and 1-4.7% have translocations. The principal cause of trisomy 21 is advanced maternal age, in which recombination errors may occur during fetal development, age-related accumulation of damaged DNA, cohesin degradation producing the premature loss of chromosomes or sister chromatids, and alterations during the spindle formation process. The paternal age has also an effect on trisomy 21, specifically during male aging, when there is higher risk of chromosomal breaking in spermatozoa. Epigenetics is also an important risk factor of trisomy 21 through changes in the DNA methylation process, histone modification and non-coding RNAs. Assisted reproductive technologies (ART) have emerged in recent years as a safe alternative for couples with fertility problems. These techniques, which include controlled ovarian stimulation (COS), in vitro fertilization (IVF), intracytoplasmic sperm injection (ICSI) and vitrification, decrease the incidence of aneuploidy in human preimplantation embryos, and are widely used. The following study aims to review and discuss the available literature on trisomy 21 in the field of assisted human reproduction.

## INTRODUCTION

The human genome is organized into 23 pairs of chromosomes, with approximately 21,200 protein-encoding genes that will determine the phenotypic and genotypic characteristics of an individual (Pertea *et al.*, 2018). Chromosomal abnormalities, involving the number of chromosomes (aneuploidy) or their structure (translocations, inversions) may translate into implantation failure, miscarriages, congenital defects, malformations, non-viable embryos, etc. (Nagaoka *et al*., 2012; Gug *et al*., 2019).

One of the main causes of aneuploidy involves chromosomal nondisjunction during meiosis as part of gametogenesis and in the first stages of embryo development (Takaesu *et al*., 1990; Pellestor *et al*., 2005; Orr *et al*., 2015). Trisomies are the most common type of aneuploidy and are responsible for approximately 50% of spontaneous abortions, of which those involving chromosomes 16, 21 and 22 are the most prevalent (Hassold & Hunt, 2001; Li *et al*., 2017).

Trisomy 21 or Down Syndrome was first described in 1866 by John Langdon Down (Down, 1995), but it was not until 1959 that Jerome Lejeune demonstrated the link between the disorder and the extra copy of chromosome 21 (Lejeune *et al*., 1959; Mills *et al*., 2011; Kurtovic-Kozaric *et al*., 2016). Down Syndrome is the most common aneuploidy in newborns, with an incidence of 1:700 - 16:10000 in live births (Weijerman & de Winter, 2010; Oster-Granite *et al*., 2011). It presents the distinctive phenotypes of the disorder, such as brachycephaly, brachydactyly, wide hands, duodenal atresia, epicanthic folds, clinodactyly of the fifth digit, flattened nosebridge, hypotonia, mental retardation, Alzheimer's disease, short stature (Roizen & Patterson, 2003), as well as characteristics that vary from case to case; like hypogonadism, cryptorchidism, cardiac malformations and leukemia (Coppedè, 2016).

Approximately, 95% of trisomy 21 babies have it because of maternal nondisjunction during meiosis, while 4% are due to a parental balanced Robertsonian translocation between chromosomes 13 or 14 and 21. The remaining 1% of Down Syndrome cases are caused by postzygotic mitotic nondisjunction (Witters *et al*., 2011). Mosaicism in Down Syndrome, in which not all cells have trisomy 21, has been reported to occur in 2-4% of cases. Mosaic Down Syndrome can be found in two categories, according to the proportion of trisomic cells present in the individual: high grade (80-90% trisomic cells) and low grade (0.1-38% trisomic cells) (Hultén *et al*., 2013).

Presently, there are several diagnostic methods with different specificities and sensibilities to detect trisomy 21 during the prenatal stage (Rink & Norton, 2016; Van Opstal *et al*., 2016; Ye *et al*., 2013); in addition to pre-implantation genetic testing for aneuploidies (PGT-A), which seeks to identify preimplantation embryos with abnormal chromosome numbers during IVF, and determine the risk factor of developing an aneuploidy-related disorder.

The present study pays particular attention to trisomy 21, its causes, incidences and risks in couples undergoing assisted reproduction procedures.

## GENETIC BASIS FOR TRISOMY 21

In Down Syndrome cases, 90-95% of patients have the full trisomy, 1.4-1.9% have mosaicism and 1-4.7% have translocations (Devlin & Morrison, 2004; Shin *et al*., 2010; Chiang *et al*., 2012; Zhu *et al*., 2013; Iwarsson *et al*., 2015; Flores-Ramírez *et al*., 2015; Karmiloff-Smith *et al*., 2016).

Full trisomy ensues during chromosome nondisjunction in meiosis I (84-86% of patients), or during chromatid segregation in meiosis II (14% of patients) (Hassold & Hunt, 2001; Patterson, 2009; Vraneković *et al*., 2012). Nondisjunction occurs when chromatids of homologous pairs fail to segregate to the opposite poles of the mitotic spindle during meiosis, and can be due to a reduction in the number of chiasmata between pairs of homologs, failure to resolve chiasmata between homologous pairs during anaphase I, and individual chromatid segregation during anaphase I, as opposed to whole chromosome segregation (Henderson & Edwards, 1968; Hassold & Hunt, 2001).

Mitotic errors after fertilization are the principal cause of embryonic mosaicism (Petersen & Mikkelsen, 2000), and can occur in 15-90% of all human embryos during the preimplantation stage (Rubio *et al*., 2007). Depending on which stage the mitotic error occurs, mosaicism can be generalized or tissue-specific (Dumanski & Piotrowski, 2012; Papavassiliou *et al*., 2015). Generalized mosaicism has its roots in a mitotic error before cellular differentiation, during the first day of embryonic development (65-70% of cases) (Wells & Delhanty, 2000; Mertzanidou *et al*., 2013). On the other hand, tissue-specific mosaicism occurs after cellular differentiation, with an incidence of approximately 50% in embryos with 4-8 blastomeres, and 10% in the inner cell mass of blastocysts (Evsikov & Verlinsky, 1998). Both generalized and tissue-specific mosaicism are originated by the same mechanisms: chromosomal nondisjunction, anaphase lagging (trisomy rescue), endoreplication or uniparental dissomy (Taylor *et al*., 2014).

In patients with mosaic Down Syndrome, the number of trisomic cells in several tissues and cells is related to the phenotypic manifestations (Modi *et al*., 2003; Papavassiliou *et al*., 2009). Respectively, mosaicism can be of high grade, in which patients have a high proportion of trisomic cells (80-90%) with distinctive Down Syndrome characteristics; or low grade, in which there is a low ratio of trisomic cells (0.1-38%), and the syndrome is not phenotypically perceptible (Hultén *et al*., 2013). The severity of genotypic and phenotypic characteristics in mosaic Down Syndrome will depend on the grade of mosaicism displayed at the cellular and histological levels, that is to say, patients with mosaic trisomy 21 could show a similar phenotype to those with non-mosaic trisomy, or even could show no phenotype at all (Papavassiliou *et al*., 2009).

In cases of trisomy 21 caused by translocations, the rearrangement involves Robertsonian rearrangements between the long arm of chromosome 21 (q21) and another acrocentric chromosome (Hultén *et al*., 2008), with chromosomes 14, 15, 22 or even the homolog of 21, being the most common (Kusre *et al*., 2015; Kalpana *et al*., 2017; Yan *et al*., 2017).

Full trisomy, mosaicism and translocations can originate from the alteration of the spindle assembly checkpoint (SAC), a protein complex that is responsible for regulating mitotic division via a feedback-control system (Musacchio, 2015). SAC specifically blocks the onset of anaphase through the inhibition of the anaphase-promoting complex/cyclosome (APC/C), until achieving chromosome attachment to the mitotic spindle at the metaphase plate. Once the spindle checkpoint requirements are met, the APC/C gets activated, causing the cleavage of cohesins that keep sister chromatids together (Pines, 2006; Lara-Gonzalez *et al*., 2012; Gorbsky, 2015). Accordingly, any disturbance to the SAC could result in the perpetuation of cell division in the presence of abnormal segregation of sister chromatids. Several other proteins and genes involved in these control mechanisms of cell division have been described, and they could be altered by factors such as aging, stress and temperature, leading to higher risk of chromosomal abnormalities (Santaguida & Amon, 2015).

## MATERNAL EFFECT ON TRISOMY 21

The process of gametogenesis in males and females has the same molecular basis, but variable predisposition to chromosomal defects. High incidence of aneuploidies are most commonly reported in oocytes, pertinent with the nature of oogenesis, which is substantially longer than spermatogenesis, occurs during both pre- and post-natal periods, and has a prolonged state of arrest during prophase I, between the fetal stage until the onset of puberty, thus increasing the probabilities of an error to occur during the segregation of homologous chromosomes (Oliver *et al*., 2008), or sister chromatids (Karmiloff-Smith *et al*., 2016).

The main causes of trisomy 21 include alterations in recombination, chromosomal nondisjunction and aging. Recombination promotes proper chromosomal orientation by the spindle apparatus to ensure subsequent separation towards opposite poles during anaphase. Absent or reduced recombination poses a risk for nondisjunction that disregards the age factor (Oliver *et al*., 2012). Nondisjunction of chromosome 21 can occur during meiosis I, meiosis II or during the first mitotic divisions of the embryo. Cases associated with nondisjunction during meiosis I are the most common, and may be associated with a lack of telomeric exchange, irrespective of maternal age (Lamb *et al*., 2005; Oliver *et al*., 2008; 2012). In contrast, in meiosis II, the number and localization of recombination hotspots (chiasmata) between chromosome 21 homologs are predisposed to abnormalities in an age-dependent manner, and enriched pericentrometric chromosomal exchanges are prevalent in older women (Oliver *et al*., 2008; Ghosh *et al*., 2009).

The molecular basis for the relationship between maternal age and predisposition to a trisomy 21 pregnancy is not clear. However, there is evidence that links the development of aneuploidies to advanced maternal age: recombination errors that occur during the fetal development of the mother, age-related accumulation of damaged DNA, cohesin degradation during dictyate that can lead to the premature loss of chromosomes or sister chromatids (Duncan *et al*., 2012) and alterations in the SAC during the spindle formation process, inevitably leading to the delay of cell division (Hauf & Watanabe, 2004; Tanaka, 2005; Touati & Wassmann, 2016). A weakened SAC would lead to the premature onset of anaphase prior to chromosomal attachment to the spindle microtubules, thus leading to potential errors in chromosome segregation (Galander *et al*., 2019). Likewise, it has been demonstrated that aging also has a negative effect in the concentration of cohesion and inhibin proteins, which are part of the SAC, and play a key role in normal cell division patterns (Duncan *et al*., 2012; Nabti *et al*., 2017).

Late motherhood is one of the main causes of infertility in women, leading to higher risks of aneuploidies during pregnancy (Allen *et al*., 2009). Trisomies are the most frequent chromosomic alterations observed in older pregnant women (Chiang *et al*., 2012). Starting at 35 years of age, the quantity and quality of oocytes begins to decrease drastically, and the risk of aneuploidies during pregnancy increases ten-fold in women over 40 years compared to women under the age of 25 years (Hassold & Hunt, 2001). Despite being the most studied factor in the nondisjunction of chromosome 21, advanced maternal age may not be the sole culprit, thus exacerbating the need for multidisciplinary approaches that may reveal links with molecular mechanisms, environmental factors, lifestyle patterns and socioeconomic conditions (Saiyed *et al*., 2018).

In *Trisomy 21 Mosaicism: We May All Have a Touch of Down Syndrome* Hultén *et al*. (2013) concluded that mosaicism of chromosome 21, in specific tissues and with variable trisomic cell proportions, is a shared characteristic in most, if not all of the general population. Considering that one may find trisomic cells in a population of fetuses with normal phenotype, the oocyte mosaicism selection model emerges, suggesting a different sexual prevalence of mosaic trisomy 21 in germinal lines, with much higher incidence in fetal ovaries than in the testes. Correspondingly, mitotic errors can emege prior to the oocyte entering meiosis, with the possibility of onset of aneuploidies in primordial follicles.

## PATERNAL EFFECT ON TRISOMY 21

Spermatogenesis is a perpetual process that extends from puberty until old age. The cycle to produce one haploid spermatozoon can last 46-76 days, resulting in minor temporal tension on the gamete and lower risk of chromosomal abnormalities (compared to oogenesis) (Heller & Clermont, 1963; 1964; Misell *et al*., 2006). Accordingly, the incidence of trisomy 21 in sperm is lower than that seen in oocytes. In addition to the discrepancy in duration of spermatogenesis and oogenesis, lower incidence of paternal trisomy 21 may also be due to the existence of a post-meiotic checkpoint in spermatogenesis, in which aneuploid spermatids or spermatozoa are arrested (Uroz & Templado, 2012).

Nondisjunction in spermatogenesis and oogenesis can be due to similar mechanisms, such as failure to resolve chiasmata between homologous chromosomes during anaphase I, absence of chiasmata between homologues that prevents appropriate chromosomal segregation, and the premature separation of sister chromatids during anaphase (Jones, 2008; Fragouli *et al*., 2011).

During spermatogenesis, the most commonly occurring abnormalities involve dissomies in secondary spermatocytes, as opposed to trisomies in primary spermatocytes. Futhermore, there is evidence of a higher number of chromosomes without recombination hotspots during meiosis I, which is associated with abnormal segregation of chromosome 21 (Oliver *et al*., 2009), and errors during meiosis II (Uroz & Templado, 2012). Secondary spermatocyte disomy is primarily caused by the absence of chiasmata of meiosis I chromosomes and errors during segregation of bivalents with reduced numbers of chiasmas, thus resulting in diploid spermatozoa and aneuploid embryos (Egozcue *et al*., 2000; Munné *et al*., 2007; Magli *et al*., 2009).

According to Iwarsson *et al.* (2015), the probability of a trisomic pregnancy caused by a spermatozoon with twice as many chromosomes 21 is less than 1 in 800 pregnancies. Contrastingly, the percentage of aneuploid embryos is higher in male-factor infertile patients (oligoasthenoozoospermia or non-obstructive azoospermia) than in infertile patients with a normal male factor (Magli *et al*., 2009).

**Figure 1:**
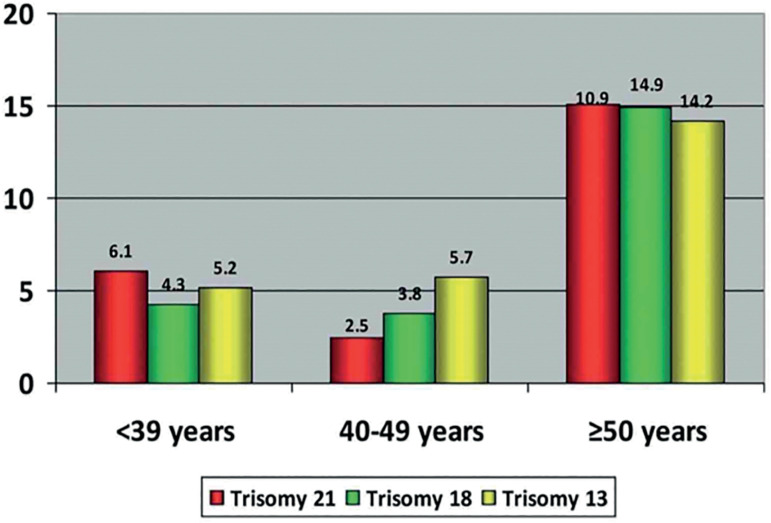
Percentages of embryos with trisomy 21, 18 and 13 in men ≤39 years, 40-49 years and ≥50 years.

Source: García-Ferreyra *et al*. JBRA Assist Reprod. 2018.

The link between paternal age and trisomy 21 has not been fully elucidated and continues to be controversial. However, studies by Carrasquillo *et al*. (2019) and Thompson (2019) showed that there was no significant association between advanced paternal age and trisomy 21; whereas Sotonica *et al*. (2016), García-Ferreyra *et al*. (2018), and Corona-Rivera *et al*. (2019) demonstrated that older fathers have a higher risk of Down syndrome pregnancies compared to younger fathers (Figure 1). Likewise, Templado *et al*. (2011) and Sotonica *et al*. (2016) revealed that paternal age is associated with an increased risk of chromosomal breaking in spermatozoa, leading to translocations commonly associated with trisomies.

When the grade of mosaicism in germline cells of male fetuses is evaluated, it is apparent that, in comparison to germline cells of the ovary, the percentage of cells with trisomy 21 is low. This finding is associated to stricter and selective control during cellular division in the testicles (Hultén *et al*., 2008; 2010), thus resulting in higher incidences of maternal influence in Down syndrome cases. On the other hand, unlike individuals with more serious cases of trisomy 21 who are considered infertile, individuals displaying mosaicism may pass on their condition or even be the direct cause of a serious trisomy 21 pregnancy. Nevertheless, more studies that illustrate the molecular mechanisms behind mosaicism in both paternal and maternal backgrounds are needed to corroborate such findings.

## EPIGENETIC EFFECT ON TRISOMY 21

Down syndrome is a gene expression disorder, involving 200-300 genes encoded in chromosome 21 (Hattori *et al*., 2000). The presence of an extra chromosome (partial or total) could lead to gene over expression (Weber *et al*., 2016). According to the "gene dosage effect" hypothesis, some of the features of Down syndrome could be directly explained by the dosage imbalance of HSA21 genes (Lyle *et al*., 2009). The extra chromosome 21 imposes a trans-acting effect in other chromosomes that cause epigenetic changes, including differential CpG methylation in specific sets of downstream target genes (Do *et al*., 2017).

Epigenetics encompasses the understanding of heritable changes in gene expression or cellular phenotype, without changing the underlying DNA sequence (Bird, 2007; Deans & Maggert, 2015), with the principal mechanisms to alter DNA expression being methylation and acetylation. DNA methylation is related to the addition of a methyl group to cytosine bases, generally located in CpG dinucleotides (Ball *et al*., 2009), by the DNA methyltransferase (DNMT) family of enzymes (DNMT1, DNMT3A and DNMT3B); whereas demethylation is mostly catalyzed by the ten-eleven translocation (TET) enzymes (TET1, TET2 and TET3) (Gensous *et al*., 2019), by action of DNA methyltransferase enzymes (DNMT1, DNMT3A Y DNMT3B) (Ciccarone *et al*., 2018a).

Gensous *et al*. (2019) have reported marked DNA methylation alterations in cells of patients with trisomy 21, being hypermethylation and genome-wide perturbance of DNA methylation the most prevalent findings. Likewise, low levels of TET enzymes, which may cause hypermethylation through decreased DNA demethylation have been reported (Jin *et al*., 2013; Ciccarone *et al*., 2018b), in addition to the disturbance of the metabolic network that produces S-adenosylmethionine (SAM); which is crucial in DNA methylation as the universal donor of the methyl group (Ball *et al*., 2009; Obermann-Borst *et al*., 2011; Obeid *et al*., 2012).

Several studies have suggested an epigenetic origin as a possible cause to the different pathologies in Down syndrome patients, involving changes in the DNA methylation process, histone modification and non-coding RNAs, which have an important role in gene expression in immune and central nervous systems diseases (Teipel & Hampel, 2006; Jones & Lane, 2013; Sailani *et al*., 2015; Wiseman *et al*., 2015). Patients with trisomy 21 show premature aging (Patterson & Cabelof, 2012), particularly in the immune and central nervous systems (Lott & Head, 2005; Teipel & Hampel, 2006). Several biological hallmarks of aging have been established, such as DNA damage buildup (López-Otín *et al*., 2013), telomere shortening (Jenkins *et al*., 2006), loss of the proteostasis network (Aivazidis *et al*., 2017), oxidative stress and mitochondrial disfunction (Pagano & Castello, 2012). These biological hallmarks, along with the epigenetics markers represent a new type of molecular marker of aging, described as a modern 'epigenetic clock' and based on DNA methylation levels (Horvath, 2013).

## ASSISTED REPRODUCTION AND TRISOMY 21

### Ovarian stimulation

In the last years, there has been a substantial increase in the age in which women decide to get pregnant, by professional or personal decisions, and this delay reduces their possibility to achieve a spontaneous pregnancy and carry a healthy baby for a full term (Munné *et al*., 1995; Nybo Andersen *et al*., 2000; Radoń-Pokracka *et al*., 2019). As a result, more and more women are turning their attention to assisted reproduction alternatives (Mills *et al*., 2011). Achieving an advanced-age pregnancy; however, this also increases the onset of chromosomal abnormalities like trisomy 21. In Europe, the last decade has been witness to a 10% increase in trisomy-21 pregnancies, all linked to higher maternal age (Loane *et al*., 2013).

Controlled ovarian stimulation (COS) with exogenous urinary or recombinant gonadotropins maximizes the number of oocytes yielded to overcome the high rate of attrition of gametes and embryos during an IVF treatment. Check (2007) suggested that COS could influence oocyte maturation and the completion of meiosis; potentially mediating chromosomal aneuploidy and mosaicism. However, all prior human studies investigating the effect of COS on embryonic aneuploidy screening a limited number of chromosomes and using fluorescent in situ hybridization analysis of blastomeres from day-3 embryos (Baart *et al*., 2007; Weghofer *et al*., 2008), have been demonstrated to have suboptimal diagnostic accuracy.

The advent of better assisted reproductive technologies has seen a rise in the use of blastocyst cultures and trophectoderm biopsies in embryology laboratories worldwide. These techniques enable natural embryonic selection through the extended culture of the blastocyst or the screening of several trophectoderm cells and 24-chromosomes. When taking into consideration the possible negative effects of COS during the aforementioned techniques, Sekhon *et al*. (2017) and Thorne *et al*. (2019) did not report a significant difference in aneuploidy rates. Likewise, Barash *et al*. (2017) showed that different doses of gonadotropins had no effect on the clinical pregnancy of women undergoing an IVF procedure. Finally, Wu *et al*. (2018) evaluated the effect of gonadotropins in two groups of patients: <35 and ≥ 35 years old, and showed that the aneuploidy rate was 40% in younger patients and 59% in older patients. Higher doses did not increase the aneuploidies rates in blastocysts within the same age group.

### *In vitro* fertilization

The frequency of aneuploidies in human preimplantation embryos generated during IVF oscillates between 56% and 84% (Fragouli *et al*., 2014), and its occurrence is related to gamete quality (Coates *et al*., 2013), maternal and paternal ages (Dailey *et al*., 1996; Fragouli *et al*., 2014; García-Ferreyra *et al*., 2015; 2018) and not with the procedure itself. Several authors have showed that the prevalence of aneuploid pregnancies in IVF procedures are similar to those observed in spontaneous pregnancies (Bingol *et al*., 2012; Pendina *et al*., 2014; Qin *et al*., 2013).

Morphological examination of oocytes during an IVF is subjective and does not allow the true assessment of the cytoskeleton and general physiology of the oocyte. Accordingly, it has been previously suggested that poor morphological quality may be related to the emergence of aneuploidies during embryonic development (Dolgushina *et al*., 2015; Lubis *et al*., 2017). Furthermore, depending on maternal age, the quality of the oocyte differs, which in turn may increase the probabilities of chromosomal abnormalities (Cimadomo *et al*., 2018). Aneuploidies have also been reported to be linked to meiotic arrest and cessation of gamete production in men, resulting in oligozoospermia or even azoospermia (Andreescu *et al*., 2016); and although trisomy 21 could have paternal origins (albeit with a lower incidence), alterations in the quality of the seminal sample may favor a higher incidence of aneuploid embryos (Coban *et al*., 2018).

Advanced age in couples may represent a major cause of infertility and a reason to explore assisted reproduction treatments to achieve pregnancy. It is well established that aneuploidy is present in embryos from infertile patients and dramatically increases with maternal age, from 73% in patients 35 years or younger, to 87% in patients 41 years or older (Fragouli *et al*., 2014). In regards to paternal age, García-Ferreyra *et al*. (2018) evaluated the aneuploidy rates in embryos obtained from egg donations, showing that men ≥ 50 years old generated significantly more aneuploidy embryos compared to younger men; and 10.9% of their embryos had trisomy 21 compared to 6.1% and 2.5% in men <39 years and 40-49 years, respectively. Therefore, advanced age in women and men significantly reduces the probability to obtain at least one euploid embryo (Ata *et al*., 2012), and increases up to 91.7% the possibility to obtain aneuploidy embryos during in IVF procedure (Su *et al*., 2016).

Assisted reproduction techniques are based on the insemination of occytes in the laboratory setting, being IVF and ICSI the most commonly sought after procedures. When comparing blastocysts obtained from IVF and ICSI treatments with normal male factor, De Munck *et al.* (2020) found similar euploid rates in both techniques (IVF: 49%; ICSI: 44%); and when evaluating the incidence of aneuploidies in chromosome 21, the authors showed that 25.8% of embryos from IVF were trisomic, compared to 20.3% of embryos from ICSI. Currently, there is no published evidence associating ICSI with the incidence of trisomy 21.

There are several factors (maternal and paternal age, gametes quality, cause of infertility, etc) that could increase the percentages of embryos with trisomy 21 generated during an IVF treatment and thus, to cause a higher prevalence of trisomy 21 babies. However, many of these trisomic embryos do not implant or are eliminated early after implantation. In cases of fertile younger women, Munné *et al*. (2017a) analyzed the external factors that affect euploidy in embryos from egg donor cycles made in different fertility centers in USA, demonstrating that the aneuploidy rates oscillate between 17-60%.

### Cryopreservation

The cryopreservation of oocytes and embryos is a technique that, due to its utility and popularity, plays an important role in assisted reproductive technology (Nagy *et al*., 2014). Ever since the first pregnancy and subsequent birth of cryopreserved oocytes and embryos (Trounson & Mohr, 1983; Zeilmaker *et al*., 1984; Chen, 1986), different protocols have emerged, differing in the type and concentration of the cryoprotectant, equilibration time, cooling rate and cryopreservation devices. Nonetheless, two of these protocols have been referenced the most in the last decades: slow freezing and vitrification (Edgar & Gook, 2012). Cryopreserving blastocysts via vitrification has improved success rates compared to slow freezing (AbdelHafez *et al*., 2010), with different survival rates (93% *vs*. 73%), implantation rates (33% *vs*. 26%) and clinical pregnancy rates (65% *vs*. 55%) (Bernal *et al*., 2008). Thus, vitrification is highly preferred and has seen a dramatic increase in assisted reproduction laboratories worldwide (Levi-Setti *et al*., 2016).

During the processes of freezing and thawing, the oocyte undergoes physical and physiological changes (Gardner *et al*., 2007) that may induce alterations in the segregation of chromosomes during meiosis II (Nottola *et al*., 2007; Noyes *et al*., 2009). Huang *et al*. (2007) demonstrated that the integrity of the mitotic spindle during chromosome alignment in cryopreserved mice oocytes was less compromised when vitrification was used. However, both slow freezing and vitrification had similar percentages of embryonic aneuploidies. In humans, it has been shown that vitrification does not increase DNA or mitotic spindle damage in oocytes, nor aneuploidies in the embryo (Forman *et al*., 2012; García *et al*., 2011; Zhang *et al*., 2015).

Oocyte vitrification and long term storage does not affect the percentage of euploid blastocysts obtained after an IVF or ICSI cycle (44.5% *vs*. 47.6% in fresh oocytes) (Goldman *et al*., 2015). In the same manner, cryopreservation enables the buildup of a higher number of oocytes to increase the number of available euploid embryos in a single cohort (Chamayou *et al*., 2017), and when vitrification is chosen due to social connotations in younger women, the risk of trisomy 21 related to maternal age decreases, as seen in cases that use oocytes obtained from a cryobank (Cobo *et al*., 2010).

## TRISOMY 21 DIAGNOSIS

The diagnosis to determine if an individual has an extra copy of chromosome 21 can be achieved in three different stages. In the postnatal stage, a confirmatory diagnosis is based on the anatomical characteristics and on the blood karyotype of the patient. In the prenatal stage, non-invasive studies may be implemented, such as hormone and DNA analysis of the mother (Ho *et al*., 2003; Yang *et al*., 2003; 2006), but a more accurate diagnosis requires invasive procedures, such as amniocentesis, chronic villus sampling or umbilical blood sampling. On the other hand, assisted reproduction techniques have enabled the cytogenetic evaluation of embryos prior to implantation, thus making embryonic selection more efficient (Munné *et al*., 2004) when compared to embryos selected by their morphological quality (up to 80% of blastocysts with "adequate" morphology can present trisomy 21) (García-Ferreyra *et al*., 2018). In the preimplantation stage and during the first days of embryonic development, a biopsy of 1 or few embryonic blastomeres can be taken (one cell at day 3 or 5-10 cells trophoblast at days 5-6) and an embryonic culture based on IVF techniques can be made.

In the last years, the preimplantation genetic testing for aneuploidies (PGT-A) with blastomere biopsy (cleavage stage) has been replaced by trophectoderm biopsy (blastocyst stage), due to problems associated with single cell analysis, both technical (e.g., high rate of amplification failure), and biological as chromosomal mosaicism, namely, the presence of cells with different karyotypes within the same embryo, seems to reach its highest level at this stage of preimplantation development (Voullaire *et al*., 2000; Bielanska *et al*., 2002). In regards to clinical outcomes, there are several studies showing the ineffectiveness and potential impairment of cleavage stage biopsy, including the review and meta-analysis of Mastenbroek *et al*. (2011) which highlighted the failure of preimplantation genetic screening when conducted by 9 chromosome FISH on biopsied blastomeres.

PGT-A with blastocyst stage biopsy strategy was reported for the first time by de Boer *et al*. (2004) and the first live births were reported by Kokkali *et al*. (2005) and McArthur *et al*. (2005), and compared to biopsy at cleavage stage, the power of this strategy resides in its higher technical and biological robustness, lower influence of procedural errors and lower impact of mosaicism on the molecular analysis. Studies by Scott Jr *et al*. (2012) comparing the ongoing pregnancy between trophectoderm and blastomere biopsy approaches, confirm the higher reliability of blastocyst stage analysis with respect to cleavage stage one (48.2% *versus* 29.2%, *p=*0.001).

In regards to amplification technologies to detect aneuploidies, many platforms are used for PGT-A as Single-Nucleotide Polymorphism (SNP) testing, array-based Comparative Genomic Hybridization (aCGH), and Next-Generation Sequencing (NGS). Each platform has its own advantages and limitations. SNP array detects segmental mutation, parental origin of monosomy and trisomy, DNA fingerprinting to prevent misdiagnosis caused by contamination, and mosaicism but it is more technically complex and needs a longer turnaround time. On the other hand, aCGH can also detect unbalanced translocation from parental Robertsonian, reciprocal translocation carriers and mosaicism, but with a lower sensitivity than NGS. NGS platforms have become increasingly popular in PGT-A because of their high throughput, their ability to precisely detect mosaicism and segmental mutation, and their capability of concomitant PGT-A and monogenic disorders. Huang *et al*. (2016) validated the NGS platform in blastocysts, demonstrating that among the aneuploid embryos identified by aCGH, re-biopsied and rechecked by NGS showed high positive (97.6%) and negative (99.6%) predictive values of aneuploidy assignment compared with the SNP array. Additionally, Friedenthal *et al*. (2018) showed that the use of NGS increased the ongoing pregnancy and live birth rates of a single frozen-thawed euploid embryo compared to aCHG.

Mosaicism in embryos is characterized by the presence of two or more genetically distinct cell lineages, typically one with a chromosome abnormality and the other showing a normal chromosome constitution (Spinella *et al*., 2018). Recent studies have suggested that diploid/aneuploid mosaicism in blastocysts is relatively uncommon (4-6%) and a preferential segregation of mosaicism did not occur in the inner cell mass of the trophectoderm (Capalbo & Rienzi, 2017; Capalbo *et al*., 2017). In regards to reproductive outcomes after transfer of mosaic blastocysts, Munné *et al*. (2017b) showed lower implantation rates (53% *versus* 70%) and higher miscarriage rates (25% *versus* 10%) compared to euploid blastocysts, but the authors also showed that 41% of mosaic embryos can produce an ongoing implantation.

Currently, non-invasive preimplantation genetic testing has become more relevant and successful, with 80-90% success rate as it is concordant with embryonic biopsies (Liñán *et al*., 2018; Capalbo *et al*., 2018) that analyses embryonic DNA present in the culture medium. However, due to its novelty, further studies are required to evaluate its concurrence with the detection of genetic abnormalities like trisomy 21 (PGT-A can confirm an aneuploidy with up to 96% concordance) (Victor *et al*., 2019).

## CONCLUSION

In humans, the trisomy 21 or Down Syndrome causes spontaneous abortions, abnormal neural development and other pathologies associated with newborn development. Depending on the severity of the genetic insult, the disorder can present itself as: a full trisomy (every cell presents a full extra copy of chromosome 21, the most common type), a translocation (one of the three chromosomes 21 is attached to another chromosome) or mosaicism (some cells have 3 copies of chromosome 21 and some are normal). However, unless a genetic study is done on the patient, telling these three types apart cannot be done phenotypically. The literature on trisomy 21 is vast and has highlighted advanced maternal and paternal ages, in addition to epigenetics, as important risk factors for the occurrence of this disorder. Accordingly, assisted reproductive technologies have emerged as important alternatives for infertile couples to achieve a healthy pregnancy. To date, there is no evidence that these technologies may increase the risk of genetic disorders like trisomy 21. Nevertheless, it is highly recommended to pair them with the appropriate genetic studies for the early detection of aneuploidies, especially in cases where the aforementioned risk factors are prevalent. Multiple platforms are used to detect aneuploidies in embryos, and the NGS is the most widely applied worldwide due to its throughput, its ability to precisely detect mosaicism and segmental mutation, and its capability of concomitant PGT-A and monogenic disorders. Finally, non-invasive preimplantation genetic testing has become more relevant and successful, with acceptable detection rates of embryonic DNA in blastocel fluid and high concordance rates; however, further studies are required to evaluate its concurrence with the detection of genetic abnormalities, such as trisomy 21.
